# Drinking Levels and Profiles of Alcohol Addicted Rats Predict Response to Nalmefene

**DOI:** 10.3389/fphar.2019.00471

**Published:** 2019-05-07

**Authors:** Jerome Clifford Foo, Valentina Vengeliene, Hamid Reza Noori, Ikuhiro Yamaguchi, Kenji Morita, Toru Nakamura, Yoshiharu Yamamoto, Rainer Spanagel

**Affiliations:** ^1^Department of Genetic Epidemiology in Psychiatry, Central Institute of Mental Health, Medical Faculty Mannheim, University of Heidelberg, Mannheim, Germany; ^2^Institute of Psychopharmacology, Central Institute of Mental Health, Medical Faculty Mannheim, University of Heidelberg, Mannheim, Germany; ^3^Neuronal Convergence Group, Max Planck Institute for Biological Cybernetics, Tübingen, Germany; ^4^Department of Physical and Health Education, Graduate School of Education, The University of Tokyo, Tokyo, Japan; ^5^Biomedical Engineering and Health Informatics Laboratory, Center for Industry-University Collaboration, Graduate School of Engineering Science, Osaka University, Osaka, Japan

**Keywords:** alcohol deprivation effect, drinkometer, nalmefene, drinking profiles, drinking risk levels, rats

## Abstract

**Background:** Pharmacotherapeutic options supporting the treatment of alcohol dependence are recommended and available but underutilized, partly due to questions about efficacy. Nalmefene, a μ-opioid receptor antagonist and partial kappa receptor agonist, is recommended for reduction of alcohol consumption, but evidence about its effectiveness has been equivocal; identifying factors which predict response will help optimize treatment.

**Methods:** The alcohol deprivation effect paradigm is a tightly controlled procedure comprising repeated deprivation and reintroduction phases, leading to increased preference for alcohol; reintroduction approximates relapse. Using a digital drinkometer system measuring high-resolution drinking behavior, we examined the effects of nalmefene on relapse drinking behavior in alcohol addicted rats. We also tested whether drinking behavior in the relapse phase prior to nalmefene administration predicted treatment response. We further examined whether longitudinal drinking behavior and locomotor activity predicted treatment response.

**Results:** Our results showed that nalmefene (0.3 mg/kg) reduced relapse-like consumption significantly (∼20%) compared to vehicle on the first 2 days of alcohol reintroduction. Examining the first 6 h of a preceded treatment-free relapse episode revealed drinking patterns clustering the rats into responders (reduction of >40%, *n* = 17) and non-responders (reduction of <40%, *n* = 7) to subsequent nalmefene treatment. During the first 6 h of the preceding relapse phase, responders consumed more alcohol than non-responders; the amount of alcohol consumed during each drinking approach was larger but frequency of drinking did not differ. Longitudinal drinking behavior and locomotor activity did not significantly predict response.

**Conclusion:** Our results suggest that nalmefene reduces alcohol intake during a relapse-like situation but effectiveness can differ greatly at the individual level. However, who responds may be informed by examining drinking profiles and rats that show high drinking levels prior to treatment are more likely to respond to nalmefene.

## Introduction

Pharmacotherapeutic options supporting the treatment of alcohol use disorder (AUD) are available and recommended for the management of AUD but are underutilized, in part due to the drugs having modest effects (Litten et al., 2018). Even the first-line treatments, acamprosate, a compound that dampens a hyper-glutamatergic state (Spanagel et al., 2005; Umhau et al., 2010; Spanagel et al., 2014), and naltrexone, a μ-opioid receptor antagonist, have only shown reductions in risk of drinking to 86% (Rösner et al., 2010a) and 83% (Rösner et al., 2010b), respectively, of the placebo rate, which does not lead to confidence in drug efficacy, motivating further research aimed at the discovery of new medications.

Nalmefene was recently approved by the European Medicines Agency as a treatment for human adults with AUD who wished to reduce their alcohol consumption but not necessarily abstain (Mann et al., 2016). Nalmefene is also a μ-opioid receptor antagonist as well as partial kappa receptor agonist (Bart et al., 2005), which is thought to have a similar mechanism of action as naltrexone (Osborn et al., 2010). Pre-clinical research has found evidence of nalmefene’s effectiveness to significantly reduce dependence-induced alcohol self-administration in rats (June et al., 1998, 2004; Calleja-Conde et al., 2016) and to be more effective than naltrexone in this respect (Walker and Koob, 2008). It is suggested that nalmefene counters alcohol-induced dysregulations of the μ- and κ-opioid receptor systems (Nealey et al., 2011).

There is also some clinical evidence for advantages of nalmefene over naltrexone (Soyka, 2016) and beneficial effects of nalmefene on endpoints such as reducing heavy drinking days and total alcohol consumption have been observed (Mann et al., 2016), particularly with an as-needed approach (Gual et al., 2013; Soyka, 2014; Francois et al., 2015). However, concerns have been raised as to the strength of this evidence and questions remain about the clinical efficacy of this drug (Palpacuer et al., 2015; Naudet et al., 2016). Response rates have been reported to vary widely, leaving significance for treatment of individual patients uncertain (Fitzgerald et al., 2016).

The identification of people who are likely to respond to nalmefene would improve treatment decisions, and identifying factors which could predict response will result in better clinical outcomes and could serve to optimize implementation of the as-needed approach (Soyka, 2014). In this respect it is important to note that the level of alcohol consumption at baseline prior to treatment may predict treatment response to nalmefene. Thus, a recent meta-analysis indicates that patients with a low or medium drinking risk level (up to 60 g alcohol per day) all failed to show any clinically relevant effect vs. placebo whereas nalmefene was modestly effective in patients with high or very high drinking risk levels (>61 g/day) (van den Brink et al., 2018) Furthermore, we have recently shown in a longitudinal preclinical study that specific drinking patterns can predict relapse behavior (Foo et al., 2017). Here we set out in a prospective study to examine if the level of alcohol consumption and drinking patterns prior to treatment are predictive of the effectiveness of nalmefene.

In the present study, we designed a longitudinal preclinical study for testing the hypothesis that drinking levels and patterns are predictive for a nalmefene response. For this purpose we used the alcohol deprivation effect (ADE) model to measure relapse-like behavior. In rats that have long-term voluntary access to alcohol followed by deprivation for several weeks, the re-presentation of alcohol leads to relapse-like drinking - a temporal increase in alcohol intake over the baseline drinking (Vengeliene et al., 2009; Spanagel, 2017). This robust phenomenon is called the ADE. This animal model has been used in numerous preclinical and translational alcohol studies and helped identifying new treatment targets with good predictive validity (Spanagel, 2009; Vengeliene et al., 2009; Spanagel, 2017). In order to precisely assess alcohol consumption and drinking patterns in our here designed prospective study we used a fully automated digital drinkometer system allowing high-resolution capture of drinking data and thus analysis of drinking profiles (Vengeliene et al., 2013). With this system we are also able to identify characteristics of alcohol drinking such as alcohol “liking” or “wanting.” An increase in alcohol “wanting” can be measured as an increased frequency of approaches to more concentrated alcohol solutions whereas alcohol “liking” is assessed by the amount of alcohol consumed per drinking approach (Vengeliene et al., 2013, 2015). With different computational approaches (Nakamura et al., 2016; Foo et al., 2017) we tested whether individual drinking levels and patterns prior to treatment are associated with nalmefene efficacy.

## Materials and Methods

### Animals

In the experiment 1, sixteen two-month old male Wistar rats were used, and in the experiment 2, twenty-four two-month old male Wistar rats (all from the breeding colony at the CIMH, Mannheim, Germany) were used. All animals were housed individually in standard rat cages (Eurostandard Type III; Ehret, Emmendingen, Germany) under an artificial 12 h/12 h light/dark cycle (lights on at 7 a.m.). Room temperature was kept constant (temperature: 23 ± 1°C, humidity: 55 ± 5%). Standard laboratory rat food (Ssniff, Soest, Germany) and tap water were provided *ad libitum* throughout the experiment. Rat body weights were measured weekly. All experiments were conducted in accordance with the ethical guidelines for the care and use of laboratory animals, and were approved by the local animal care committee (Regierungspräsidium Karlsruhe, Germany).

### Drugs

Alcohol drinking solutions were prepared from 96% ethanol (Sigma-Aldrich, Germany) and then diluted with tap water. Nalmefene (Lundbeck, Denmark) was dissolved in 0.9% saline. The solution was freshly prepared and injected as a volume of 1 ml/kg subcutaneously (s.c.). Control experiments were performed following administration of saline.

### Drinkometer System

The experiments were performed using a computer-monitored Drinkometer system (TSE Systems, Bad Homburg, Germany), which enables continuous long-term monitoring of liquid consumption by amount and time in a standard rat home cage (Eurostandard Type III). The system is equipped with four drinking stations to allow liquid choice. Each drinking station consists of a glass vessel containing the liquid and a high precision sensor for weighing the amount of liquid removed from the glass vessel. Spillage and evaporation are minimized by using special bottle caps. The whole system is mounted to a custom-made free-swinging steel frame in order to avoid any environmental disturbances (see also Vengeliene et al., 2013). The weight of each vessel is measured in 200 ms steps and saved in 1 s steps, and ultra-high resolution changes in volume are detected down to 0.01 g. For experiment 1, the weight of drinking vessels was recorded in 5-min intervals, and for experiment 2, the sampling interval was set at 1-min, giving per minute values of solutions consumed.

### Long-Term Voluntary Alcohol Consumption With Repeated Deprivation Phases

The ADE is a tightly controlled experimental procedure used to model excessive relapse-like drinking in rodents (Vengeliene et al., 2013). The procedure begins with a long-term (8 weeks) baseline period of voluntary alcohol consumption in a four-bottle free-choice paradigm in which rats are continuously presented with water and three different concentrations of ethanol (5, 10 and 20%). This baseline period is followed by a two-week deprivation period, after which alcohol is reintroduced and the ADE, which is characterized by robust increases in alcohol intake and preference for stronger solutions, is observed. Subsequent deprivation (2 weeks long) and reintroduction phases (4–6 weeks long) are randomly introduced, resulting in an increased preference for alcohol. Thus, drinking patterns during the ADE represent an important target for understanding both relapse mechanisms and investigating effects of drug treatment.

### Locomotor Activity Measurements

Rat locomotor activity was monitored by use of an infrared sensor connected to a Mouse-E-Motion recording and data storing system (Infra-e-motion, Henstedt-Ulzburg, Germany). The device was placed above each cage so that the rat could be detected at any position inside the cage. The device sampled every second and the sensor could detect rat body movements at least 1.5 cm from one sample point to the successive one. For the experiment 1, monitoring of locomotor activity started 4 days before the drug treatment procedure and was continued for two more post-treatment days. The percentage of each rat’s locomotor activity during and after treatment days was calculated by using the “before treatment” activity data as a reference. For the experiment 2, locomotor activity was measured continuously and recorded every minute during the initial 8-week baseline drinking period, as well as first, second, fifth and sixth ADE periods (see also Foo et al., 2017).

### Experiment 1

Nalmefene treatment was introduced at the end of the 8th alcohol deprivation. In order to study the effects of nalmefene, rats were divided into two groups (*n* = 8) in such way that the mean baseline intake of water and 5, 10, and 20% of alcohol solutions was approximately the same in each group. Baseline drinking was monitored daily for one week. After the last day of baseline measurement, the alcohol bottles were removed from the cages leaving the animals with free access to food and water for 19 days. Thereafter, each animal was subjected to a total of 5 s.c. injections (starting at 7 p.m. with 12 h intervals) of either vehicle or nalmefene (0.3 mg/kg, please note that a prior experiment employing the same experimental paradigm using 0.01 and 0.1 mg/kg did not produce significant effects, this data is not shown). The alcohol bottles were reintroduced after the second drug administration (at ∼9 a.m. on the 20th day of alcohol deprivation). Each rat’s body weight was recorded 24 h before the first injection and 12 h after the last injection.

### Experiment 2

In order to explore whether or not the response to nalmefene could be predicted by certain drinking patterns or behavior, we conducted a second experiment with a larger sample and higher resolution assessments, examining the ADE prior to nalmefene administration. In this experiment, nalmefene was injected s.c. to all rats at the end of the 6th two-week long alcohol deprivation. Each animal was subjected to a total of 5 s.c. injections (starting at 7 p.m. with 12 h intervals) of nalmefene (0.3 mg/kg). Similarly to the first experiment, the alcohol bottles were reintroduced after the second injection. Each rat’s body weight was recorded 24 h before the first injection and 12 h after the last injection.

### Data Analysis

Data on total daily ethanol intake, water intake and locomotor activity from experiment 1 was analyzed using a two-way analysis of variance (ANOVA) with repeated measures (factors were: between subjects – treatment group, and within subjects – day). For the analysis of locomotor activity, only the data from the dark phases was used. Whenever significant differences were found, *post hoc* Student Newman–Keuls test was performed. Data analysis regarding the effects of treatment on the change in the rat body weight was performed using either a one-way ANOVA or independent two-tailed *t*-test.

The effects of nalmefene from experiment 2 were first examined comparing the first 3 days of the 5th (ADE5) and 6th (ADE6) ADEs. Two-way ANOVA with repeated measures was used. The first 6 h after reintroduction of alcohol were analyzed in greater detail as this is the period where the strongest ADE occurs. This data was analyzed using two-way ANOVA with repeated measures (factors were: between subjects – ADE, and within subjects – time). To quantify response to nalmefene treatment, total alcohol consumption (intake of pure ethanol in g per kg of body weight in 6 h) was compared across ADE5 and ADE6 to give a % reduction score: Response = (Consumption_ADE5_ – Consumption_ADE6_)/Consumption_ADE5_. After examining the response distribution it was found that two clusters emerged above and below the mean. We thus used the mean to classify response of rats to nalmefene: those below the mean response level were classified as “Non-responders” and those above it were classified as “Responders.” To test whether established drinking levels and profiles existing prior to nalmefene administration could inform response to nalmefene, we calculated during ADE5 during the first 6 h of alcohol re-exposure: (Litten et al., 2018) total alcohol intake (Spanagel et al., 2005) approach frequency (i.e., average number of approaches) for each alcohol solution and (Umhau et al., 2010) approach size (i.e., amount of alcohol consumed per drinking approach). To characterize the effects of nalmefene in Responders and Non-responders, we also looked at the drinking profiles in ADE6. Comparisons between Responders and Non-responders were made using Welch’s ANOVA given unequal sample sizes. The chosen level of significance was *p* < 0.05.

Longitudinal locomotor activity was examined after classification of Responders and Non-Responders. It was examined whether locomotor activity patterns differed between the two groups during ADE5 and ADE6, as well as during initial baseline and deprivation periods during which addiction-like behavior was established. Following our previous work (Foo et al., 2017) we examined whether weekly activity, representing stable locomotor patterns (6-day intervals, not including the day when experimenters entered the room), was informative with respect to response status to nalmefene. Based on per minute activity counts, we calculated local statistics for locomotor activity over the week for each rat. Mean per minute activity, as well as variance and skewness of the weekly locomotor distribution were examined. Differences in mean activity could indicate psychomotor agitation/retardation, while variance characterizes variability of activity and skewness represents intermittency of activity; shown to be biomarkers able to characterize disease states (Kim et al., 2013; Nakamura et al., 2016). Furthermore, circadian amplitude was also calculated for the weekly locomotor data. Briefly, the continuous wavelet transform, which can be used to characterize periodic patterns, was applied to the weekly locomotor data. The power of the continuous wavelet coefficient for the frequency 1 cycle/day, or circadian amplitude, was extracted (see Foo et al., 2017 for further detail). Repeated measures ANOVA was used to test for differences between longitudinal group trajectories.

## Results

### Experiment 1

**FIGURE 1:**
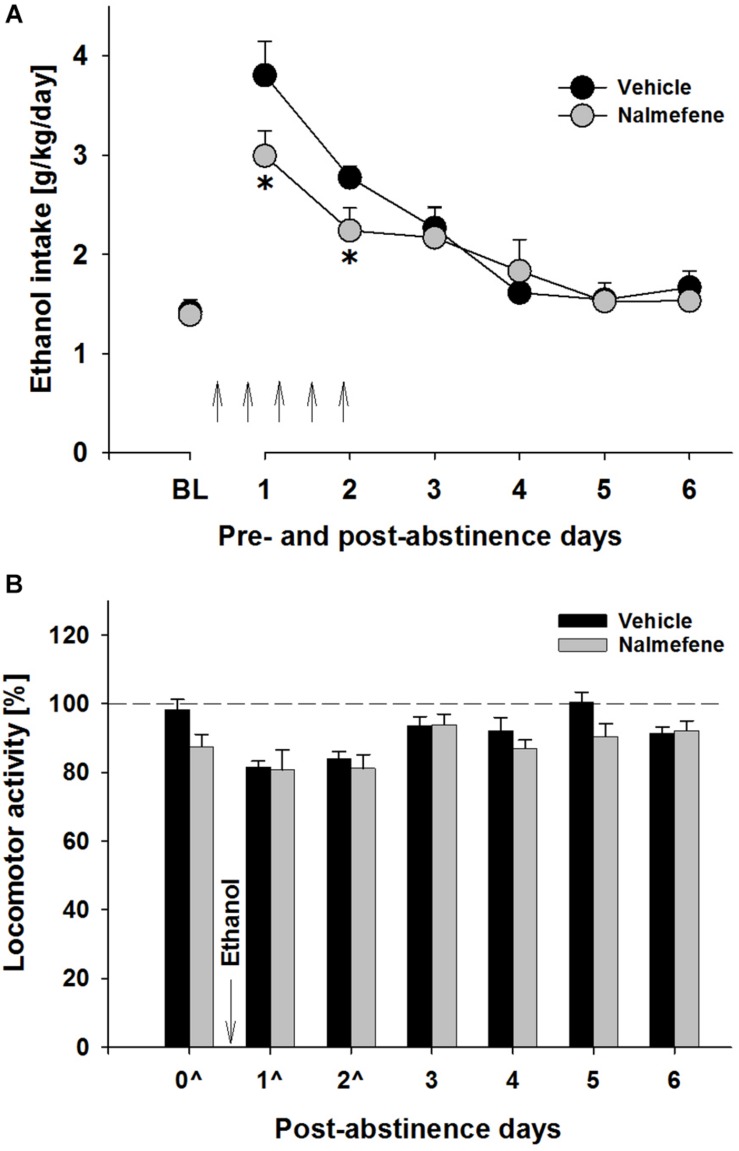
Total ethanol intake (g/kg/day) before and after an alcohol deprivation period of 3 weeks **(A)** and locomotor activity of the last abstinence day and the first post-abstinence days **(B)**. Arrows indicate the administration of vehicle (*n* = 8) and 0.3 mg/kg of nalmefene (*n* = 8). A total of 5 injections were given (starting at 7 p.m. with 12 h intervals), the first injection was given 12 h before post-abstinence drinking begun. The average of the last 6 days measurements of ethanol intake is presented as the baseline drinking (BL). Locomotor activity is shown as 12-h post-injection intervals of the animals’ active phase. The percentage of each rat’s locomotor activity during and after treatment days was calculated with respect to basal activity prior to treatment (dashed line). Data are presented as means ± S.E.M. *indicates significant difference from the vehicle control group, *p* < 0.05.

Following the re-introduction of alcohol solutions after a period of abstinence, the vehicle treated group showed a typical increase in alcohol consumption, indicating the occurrence of an ADE. This increase was not different from that observed during the first deprivation periods (data not shown). Hence, a two-way ANOVA for repeated measures revealed a general increase in alcohol intake after an eight deprivation phase as compared to basal drinking [factor day: *F*(6,84) = 41.8, *p* < 0.0001] (Figure 1A). Analysis of data also showed that nalmefene treatment significantly reduced alcohol intake during the first post-abstinence days when compared to intake by vehicle treated animals [factor treatment group × day interaction effect: *F*(6,84) = 2.5, *p* < 0.05]. This treatment did not cause loss of body weight, however, animals from the nalmefene treatment group did not gain as much body weight as the vehicle treated rats [vehicle: +1.4% and nalmefene: +0.7%, factor treatment group *t*(14) = 2.5, *p* < 0.05]. Stronger doses might increase response, but may also result in unwanted effects. Nalmefene treatment had no effect on either water intake [factor treatment group × day interaction effect: *p* = 0.84] (data not shown) or home-cage activity of rats measured as total number of movements during their active phase [factor treatment group × day interaction effect: *p* = 0.71] (Figure 1B), demonstrating that this drug is well tolerated.

### Experiment 2

**FIGURE 2:**
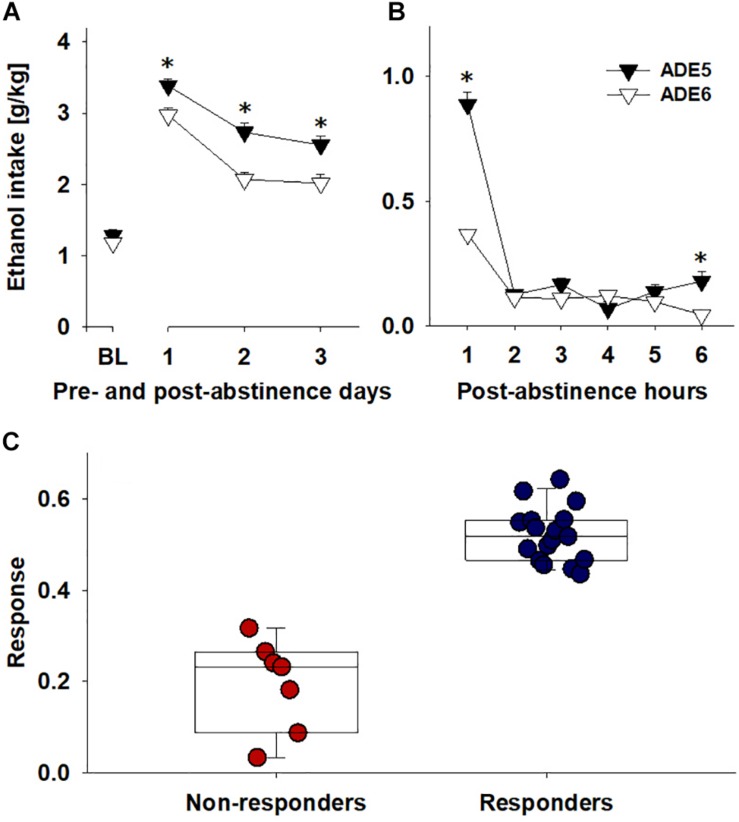
Comparing ADE5 and ADE6 for response to nalmefene: ethanol intake for **(A)** baseline and the first three post-abstinence days and **(B)** first 6 h post-abstinence. **(C)** To quantify response to nalmefene treatment, total alcohol consumption (intake of pure ethanol in g per kg of body weight in 6 post-abstinence hours) was compared across ADE5 and ADE6 to give a % alcohol consumption reduction score: Response = (Consumption_ADE5_ – Consumption_ADE6_)/Consumption_ADE5_. Examination of the distribution of reduction of ethanol intake during the first 6 h allowed classification of rats into two groups, Responders (blue, rats above the mean response level, *n* = 17) and Non-responders (red, rats below the mean response level, *n* = 7). Data are presented as means ± S.E.M. *indicates significant difference from ADE6, *p* < 0.05.

When comparing the first 3 days of the regular ADE period (ADE5) preceding the ADE period with nalmefene administration (ADE6 – all 24 animals received s.c. nalmefene injections), it was observed that for each day and for the whole 3 day period, rats consumed less total alcohol during ADE6 [factor ADE: *F*(1,46) = 15.5, *p* < 0.001 and factor ADE × time interaction effect: *F*(3,138) = 3.6, *p* < 0.05] (Figure 2A). While statistically significant, this effect was modest (i.e., an average of 18.6% decrease). As mentioned above, to better quantify the drinking behavior, we also analyzed drinking during the first 6 h of the ADE, since during the first hours after alcohol reintroduction the ADE is the largest with effects tapering off quickly over time (Vengeliene et al., 2009, 2013; Foo et al., 2017). Hence, the overall decrease of hourly consumption between ADE5 and ADE6 was significantly different [factor ADE: *F*(1,46) = 81.0, *p* < 0.0001], and this difference was most pronounced during the first hour of alcohol re-exposure [factor ADE × time interaction effect: *F*(5,230) = 28.6, *p* < 0.0001] (Figure 2B). The average Response (i.e., a % reduction score described in Data Analysis) during the first 6 h was 42.6%. After examining the distribution of responses for all rats, the rats were classified into two groups: those below the mean were classified as “Non-responders” and those above it were classified as “Responders” (Figure 2C).

#### Drinking Profiles During ADE5

**FIGURE 3:**
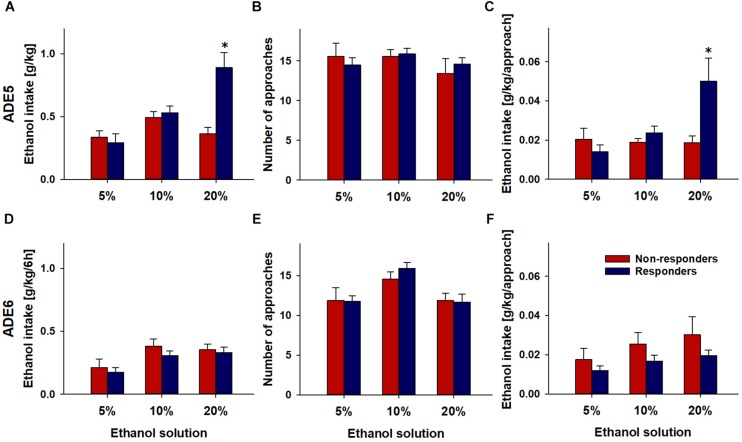
Drinking profiles of rats (*n* = 24) during the first 6 post-abstinence hours of ADE5 **(A–C)** and ADE6 **(D–F)**. Repeated administration of 0.3 mg/kg of nalmefene was performed during ADE6. Total ethanol intake **(A,D)**, number of approaches **(B,E)** and amount of ethanol consumed per approach **(C,F)** for each alcohol solution is shown in rats above the mean response level (Responders, *n* = 17) and below the mean response level (Non-Responders, *n* = 7) to nalmefene treatment. Data are presented as means ± S.E.M. *indicates significant difference from Non-responders, *p* < 0.05.

Drinking behavior during ADE5 was analyzed to see whether drinking levels and patterns of rats could predict effects of nalmefene. Examining consumption patterns in Responders and Non-Responders during ADE5 revealed that overall, responders had significantly higher alcohol consumption levels during the first 6 h of ADE5 [*F*(1,20.5) = 39.8, *p* < 0.001] (Figure 3A). This effect was solely driven by higher consumption of 20% alcohol during this time [*F*(1,20.9) = 16.5, *p* < 0.001]. Intake of 5 and 10% alcohol did not differ between Responders and Non-responders (Figure 3A). Frequency of approaches to the alcohol bottles – which we have used as an indication of “alcohol wanting” in our previous work (Vengeliene et al., 2013, 2015) did not differ significantly between groups neither for each alcohol solutions nor for alcohol accesses in total (Figure 3B). Average total alcohol approach size was larger in Responders [*F*(1,21.5) = 6.6, *p* < 0.05], an effect which appears to have been driven by higher approach size of 20% solution [*F*(1,18.6) = 6.6, *p* < 0.05] (Figure 3C). The amount of alcohol that is consumed during a drinking approach has been defined in our previous studies as an indication for “alcohol liking” (Vengeliene et al., 2013, 2015). This finding leads to the conclusion that nalmefene responders show higher preceding drinking levels which are mainly driven by alcohol liking.

#### Drinking Profiles During ADE6 (Nalmefene Treatment)

During the first hours of ADE6, total alcohol intake during the first 6 h after alcohol re-exposure in Responders was lower than that in Non-responders [*F*(1,12.9) = 6.9, *p* < 0.05] (data not shown). This appears to have been a result of an additive effect across solutions; although all solutions showed reduced consumption, individual solution approach size did not statistically significantly differ (5%: *p* = 0.41; 10%: *p* = 0.21; 20%: *p* = 0.30). No significant effects of nalmefene were observed on approach frequency [*p* = 0.73] (Figures 3D–F).

#### Longitudinal Locomotor Activity

**FIGURE 4:**
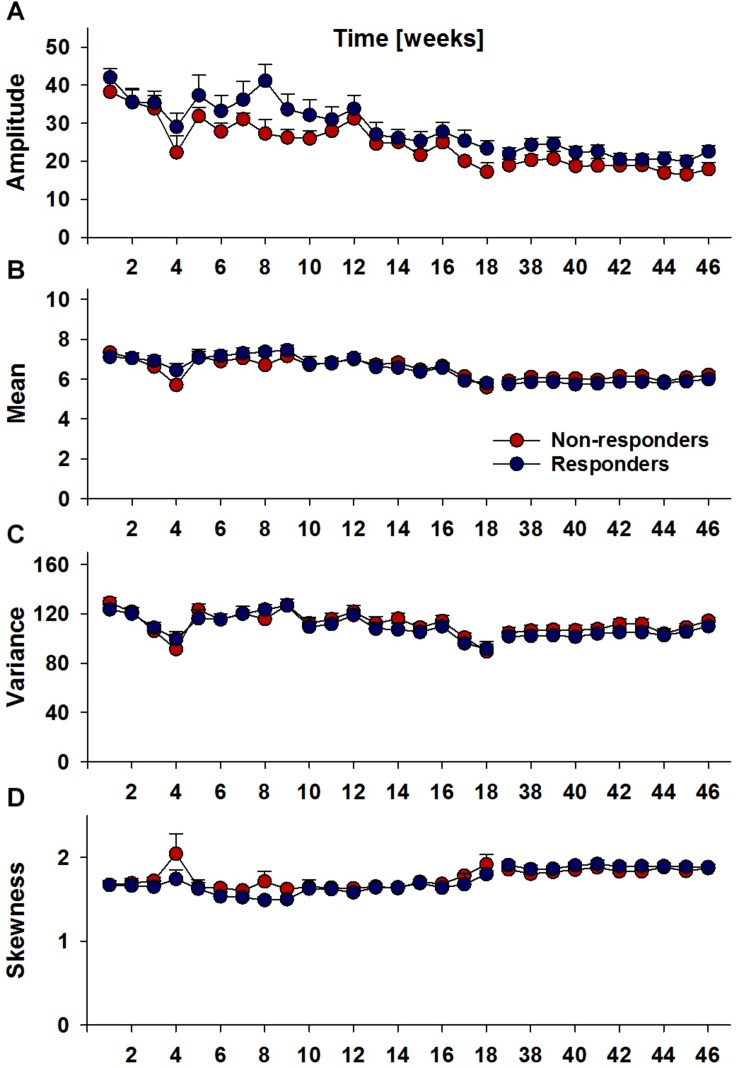
Longitudinal locomotor activity parameters, circadian amplitude **(A)**, mean per-minute locomotor activity **(B)**, variance **(C)** and skewness **(D)** per week in Responders (*n* = 17) and Non-Responders (*n* = 7). Analyses did not reveal significant group differences.

Recently, we were able to show that alterations in locomotor activity patterns; especially, instability of circadian rhythms (Foo et al., 2017), can be predictive of future relapse behavior. Therefore, we also examined locomotor activity patterns for Responders and Non-Responders. Descriptively, comparison of longitudinal circadian amplitude trajectories was suggestive of differences between Responders and Non-responders, but this main effect did not reach significance (i.e., *p* = 0.18) (Figure 4A). For mean, variance and skewness measures of locomotor activity patterns again no differences were observed between Responders and Non-responders (Figures 4B–D).

## Discussion

Our study demonstrated that nalmefene treatment reduced relapse-like alcohol consumption in male rats during the first post-abstinence days in a four-bottle free-choice setting. Nalmefene was the most effective in rats which consumed greater amounts of highly concentrated alcohol per drinking approach prior to drug treatment. Nalmefene treatment did not change water intake and locomotor activity compared to vehicle treatment, demonstrating the selectivity of the tested compound toward alcohol consumption.

The *in vivo* pharmacology of nalmefene is suggested to be similar to naltrexone (Osborn et al., 2010; Spanagel and Vengeliene, 2013), acting on opioid receptors and it shares the ability to reduce the subjective “high” feeling after alcohol consumption (i.e., “alcohol liking”) (Drobes et al., 2004; Hendershot et al., 2017). In the present experiments, the effects of nalmefene (reduction of approximately 20% in alcohol consumption) were comparable with that of other opioid antagonists given under similar experimental conditions (Hölter and Spanagel, 1999).

Like other medications for relapse prevention, nalmefene is only modestly effective with many patients failing to benefit from this treatment. Especially, inter-individual variability may play a role for the effectiveness of nalmefene (Fitzgerald et al., 2016). As such, it is important to identify potential high responders, so that the maximum benefits can be derived from the drug. The distribution of response to nalmefene in our study suggests a role for inter-individual differences; while most rats experienced reductions in alcohol consumption (ranging from 3.4 to 64.2% in the first 6 h of ADE) as a result of treatment, rats were also clearly separable into better and poorer responding groups. The results of the present investigation suggest that drinking profiles and alcohol consumption levels may be able to inform drug response. We observed that those rats that exhibited more pronounced response to nalmefene had consumed more alcohol, especially of a higher concentration, during the preceding “regular ADE,” potentially suggesting that better responders have a stronger preference for alcohol. This finding on the preclinical level is in line with a recent meta-analysis that indicates that patients with a low or medium drinking risk level (up to 60 g alcohol per day) all failed to show any clinically relevant effect vs. placebo, whereas nalmefene was modestly effective in patients with high or very high drinking risk levels (>61 g/day) (van den Brink et al., 2018). Thus, individuals that show high baseline alcohol consumption levels prior treatment are more likely to respond to nalmefene. This is an important conclusion, since patients that have high or even very high drinking risk levels are the ones who experience the most severe health consequences (Rehm et al., 2018).

We also observed that the effects of nalmefene were driven by reduction of alcohol consumption per drinking approach, while no reductions in frequency of approaches to alcohol bottles were observed. Thus, our results appear to inform the distinction between “liking” and “wanting,” which has been an important topic in the field of addiction (Robinson and Berridge, 2003; Spanagel, 2009). Finding that approach size during a drinking episode, but not frequency was reduced during ADE supports the idea of a separation of “wanting” and “liking” (Berridge and Aldridge, 2008; Vengeliene et al., 2013, 2015), which is consistent with the idea that the neural systems responsible for ethanol “wanting” are different from those that mediate the hedonic “liking” effects of alcohol (Spanagel, 2009); and drug treatments may differentially target these mechanisms. For example, it has been observed that at higher levels of alcohol craving, drinking was reduced at a significantly greater rate with naltrexone as compared to acamprosate (Richardson et al., 2008). The present results support the idea that the mechanism of action of nalmefene is on the hedonic value of ethanol; while rats continue to approach the bottles with the same frequency, they drink less as the alcohol is rendered less appetitive by the drug. This is in line with recent neuroimaging evidence of a nalmefene-caused reduction in “reward anticipation” in striatal regions (Quelch et al., 2017). This hedonic “liking,” is thought to be governed by opioid neurotransmission in the rostro-dorsal quarter of the medial nucleus accumbens shell (Pecina, 2008).

In terms of longitudinal locomotor activity, descriptively, the measure of circadian amplitude suggested that responders may have higher circadian amplitudes than non-responders, but these group differences did not reach significance. These analyses did not yield evidence of overarching longitudinal differences between groups; differences may not extend to long-term behavioral patterns and may be restricted to local relapse periods.

It should be recognized that due to the efficacy of the drug, the number of responders and non-responders were necessarily unbalanced; larger sample sizes will be needed to further test these results, and it is expected that increased power will improve the ability of these approaches to identify differences between groups, informing drug treatment response. It should also be noted that the present definition of response to nalmefene is based on short-term effects at a specific dosage. Long-term administration may reveal different response patterns. It is possible that not only response but also sensitivity is reflected in the present findings, and further research is needed to clearly delineate underlying mechanisms. Determining the relationship between ethanol consumption and effects on opioid systems may lead to improved target selection and the development of a different class of opioid drugs.

The present findings were made possible through the use of a drinkometer which acquired continuous longitudinal data. In humans, it is difficult to reach the time resolution and accuracy offered by the drinkometer and efficacy measures such as quantity and frequency of drinking have thus far only been acquired in a comparatively limited fashion (much research examines abstinence rates and or number of binge days using retrospective self-reports or other cross-sectional methods). In the emerging era of Information and Communication Technologies (ICT), acquisition of this data is becoming possible using mobile and wearable technologies and an ambulatory assessment approach (Trull and Ebner-Priemer, 2013), and is likely to be key to further characterize treatment response and help to better identify and quantify effective treatment time courses. Furthermore, to improve translatability of findings to clinical applications, research needs to be extended in the direction of including female animals.

Prospective clinical studies are now needed to test if the drinking profiles identified here and alcohol drinking risk levels are indeed predictive for the efficacy of nalmefene. If “baseline drinking profiles” can be assessed through the detailed and rigorous collection of data in clinical settings is expected to be important to inform the targeted use of not only nalmefene in the recommended “harm-reduction,” “as needed” approach, but other drugs and types of treatments.

## Author Contributions

RS and VV designed the study, interpreted the findings, and wrote the manuscript. JF analyzed the data, interpreted the findings, and wrote the manuscript. VV performed the experiments and analyzed the data. HN, IY, KM, TN, and YY analyzed the locomotor activity data. All authors approved the final version of the manuscript.

## Conflict of Interest Statement

The authors declare that the research was conducted in the absence of any commercial or financial relationships that could be construed as a potential conflict of interest.
